# Auxin–rhizomicrobiome interactions as a driver of climate-resilient root architecture: a conceptual framework for sustainable agriculture

**DOI:** 10.3389/fpls.2026.1839892

**Published:** 2026-09-15

**Authors:** Abdul Mumeen Iddrisu, İnanç Özgen

**Affiliations:** 1 Graduate School of Natural and Applied Sciences, Firat University, Elâzığ, Türkiye; 2 Faculty of Engineering, Firat University, Elâzığ, Türkiye

**Keywords:** abiotic stress, auxin signaling, plant-microbe interactions, rhizosphere microbiome, root system architecture, sustainable agriculture

## Abstract

Abiotic stresses such as drought and salinity represent major constraints to global crop productivity, necessitating innovative strategies for enhancing plant resilience. As a critical regulator of root system architecture, auxin (specifically indole-3-acetic acid (IAA)) functions as a vital cross-kingdom signaling molecule that mediates dynamic interactions between plants and auxin-synthesizing rhizosphere microorganisms. This review explores the role of auxin as a cross-kingdom signaling molecule mediating interactions between plants and the rhizosphere microbiome. Auxin-producing plant growth-promoting rhizobacteria (PGPR) such as *Pseudomonas*, *Bacillus*, and *Azospirillum* can modify root system architecture by stimulating lateral root formation, root hair development, and root elongation. These structural changes enhance soil exploration, improving water and nutrient acquisition under stress conditions. In addition, root exudates released by plants recruit beneficial microbial communities, establishing a feedback loop that stabilizes plant–microbe interactions in the rhizosphere. While previous studies have largely treated plant hormonal signaling and rhizosphere ecology as separate domains, this review bridges these silos by proposing the Auxin–Rhizomicrobiome–Root Architecture (ARRA) model; an integrative framework demonstrating how microbial hormone production and plant signaling networks jointly program adaptive root traits under climate stress. Ultimately, the ARRA framework provides a conceptual and practical blueprint for deploying auxin-producing bioinoculants and engineered rhizomicrobiome consortia, offering a scalable strategy to enhance crop resilience and sustainable food security under accelerating climate scenarios.

## 1 Introduction

Abiotic stress, especially moisture and temperature related ones, is increasingly becoming a global concern for crop producers and policy makers (IPCC, 2022). Consequently, navigating the intersection of climate instability and global food insecurity has forced researchers and policymakers to prioritize the development of sustainable, climate-resilient cropping systems (O’Sullivan, 2023). Research has established that, among the various physiological mechanisms involved in plant adaptation to environmental stress, the health of the root system and its architecture play critical role in a plant’s capacity to explore and acquire limited soil resources (Ranjan et al., 2024; Zinta et al., 2022). This is because they determine a plant’s capacity to explore soil resources under limiting conditions. According to Santner et al. (2009), phytohormones such as auxin are among the most critical drivers of root system development. Auxin regulates a wide range of physiological processes including cell elongation, apical dominance, vascular differentiation and root development (Taiz et al., 2015). In the context of root biology, auxin governs key developmental processes such as lateral root initiation, root hair formation and root elongation, thereby shaping the overall architecture of the root system (Sabagh et al., 2021). These auxin-mediated processes occur in interaction with other phytohormones in enabling plants adjust their root structure in response to environmental signals (Sabagh et al., 2021; Taiz et al., 2015). Despite extensive advances in plant physiology and microbiome research, a unified framework integrating auxin signaling with rhizosphere microbial dynamics under climate stress remains lacking. Addressing this gap is essential for developing next-generation, microbiome-assisted strategies for sustainable and climate-resilient agriculture.

Crucially, recent advances in rhizosphere biology have dismantled the view of auxin as an exclusively endogenous plant hormone, demonstrating that it functions as a shared, cross-kingdom signaling currency actively synthesized by diverse rhizosphere microorganisms (Jan et al., 2024; Xu et al., 2023, 2025; Zhang et al., 2025). Many plant growth-promoting rhizobacteria (PGPR) and fungi are capable of synthesizing IAA (Zhang et al., 2025). Microbial auxin production can significantly influence plant growth by altering root morphology and stimulating the formation of lateral roots and root hairs (Xu et al., 2023). The rhizosphere, the dynamic soil zone directly influenced by root exudates, serves as the primary ecological niche where host plants engage in complex biochemical exchanges with symbiotic fungi, mycorrhiza, and plant growth-promoting rhizobacteria (PGPR) (Kuzyakov and Razavi, 2019). This could range from their interactions with rhizobacteria and mycorrhiza to other plants’ roots. Plants release an array of root exudates, including sugars, amino acids, organic acids and secondary metabolites, which shape the composition and activity of microbial communities (Chen and Liu, 2024; Ma et al., 2022; Martins et al., 2026). On the other hand, rhizosphere microorganisms produce certain compounds such as phytohormones, enzymes as well as signaling molecules that influence plant physiology (Ranjan et al., 2024). In this context, auxins play critical roles in mediating the interactions between the plant and rhizosphere microbiome.

Cumulating evidence underscores the foundational role of auxin signaling in mediating plant abiotic stress responses (Jing et al., 2023; Salehin, 2024), with recent breakthroughs highlighting how interactions with the rhizosphere microbiome dynamically modulate root system architecture (RSA) to amplify this tolerance (Mal and Panchal, 2024). While plants actively produce endogenous auxins to regulate growth, external auxins synthesized by rhizosphere microorganisms can supplement this pool, serving as a critical environmental trigger that modulates root development (Xu et al., 2023; Zhang et al., 2025). In a beneficial scenario, such interactions may lead to the formation of much deeper and branched root systems, as such, improves soil exploration. Effective soil exploration results in enhanced plant tolerance to environmental stresses such as drought and salinity. Furthermore, these auxin-driven changes in RSA actively reshape the rhizosphere microenvironment, setting the stage for long-term beneficial plant–microbe associations (Martins et al., 2026). Despite growing recognition of the importance of plant–microbiome interactions in agriculture, the integrated role of auxin signaling within this system remains insufficiently explored (Jing et al., 2023; Salehin, 2024). Unraveling the deep biochemical interconnectedness of plant hormonal signaling and rhizosphere ecology using contemporary multi-omics is essential. Understanding how microbial auxin production interacts with plant auxin pathways to regulate RSA provides valuable insights for developing microbiome-assisted strategies for sustainable crop production.

In this context, this paper explores the role of auxin-mediated interactions between plants and their rhizosphere microbiome in shaping root system architecture as it relates to enhancing abiotic stress tolerance. This paper, thus, proposes a conceptual framework in which auxin serves as a central mediator linking rhizosphere microbial activity with plant adaptive responses. Climate change-driven stress conditions necessitate integrative approaches combining plant physiology and microbiome engineering. Such approach may lead to the development of novel agricultural production systems capable of sustainably improving crop performance in climate-stressed environments.

## 2 Auxin biology and root system architecture

Auxin serves as an important regulator of plant growth and development, controlling a wide range of physiological and developmental processes throughout a plant’s life cycle (Taiz et al., 2015). Among the naturally occurring auxins, indole-3-acetic acid (IAA) is the most abundant and biologically active form in higher plants (Jing et al., 2023). By directly directing cellular division and expansion, auxin plays an essential role in structural morphogenesis. Chief among these processes is the auxin-mediated modulation of root system architecture (RSA)—the spatial configuration of roots within the soil profile encompassing traits such as primary root length, lateral root density, root angle, and root hair development (Gupta et al., 2023; Orozco-Mosqueda et al., 2023; Taiz et al., 2015; Weihs et al., 2024). Recent advances in artificial intelligence-based phenotyping have further improved the quantitative analysis of root system architecture under stress conditions (Weihs et al., 2024). Physiologically, auxin is one of the major phytohormones, and arguably the most important, recognized as the primary regulators of RSA (Kou et al., 2022). Through a mechanism of influencing root cell division, elongation and development, auxin enables plants to modify their root system in response to environmental signals.

At the molecular level, auxin signaling operates through a well-characterized regulatory pathway involving AUX/IAA proteins, auxin response factors (ARFs) and auxin transport proteins (Taiz et al., 2015). According to Enders and Strader (2015), the absence of auxin, triggers AUX/IAA proteins to repress the activity of ARF transcription factors thereby preventing the expression of auxin-responsive genes. On the other hand, when auxin levels increase, the hormone promotes the degradation of AUX/IAA repressors, allowing ARF transcription factors to activate genes which regulate cell division and differentiation. This signaling cascade ultimately controls the developmental processes that shape root morphology. Another essential component of auxin regulation is polar auxin transport, which establishes hormone gradients within plant tissues (Taiz et al., 2015). Auxin distribution in roots is largely controlled by specialized membrane transport proteins. Enders and Strader (2015) indicated that the members of the PIN-FORMED (PIN) and ATP-BINDING CASSETTE SUBFAMILY B (ABCB) protein families facilitate the efflux of IAA, whereas members of the AUXIN RESISTANT1/LIKE AUX1 (AUX1/LAX) transporters facilitate IAA uptake. These transport systems create localized auxin maxima that determine sites of lateral root initiation and root meristem activity (Enders and Strader, 2015). Consequently, the spatial distribution of auxin within the root system plays a decisive role in defining the pattern and extent of root branching (Enders and Strader, 2015).

As stated earlier, auxin-mediated root development is linked with environmental and adaptation demand factors. Plants frequently modify their root architecture in response to external stimuli such as soil moisture, nutrient availability and salinity (Kuzyakov and Razavi, 2019; Weihs et al., 2024; Zinta et al., 2022). For instance, in a meta-Analysis, it was observed that under moisture stress conditions, plants tend to develop deeper root systems that enable access to water in deeper layers of the soil profile (Kou et al., 2022). Similarly, nutrient deficiencies can stimulate lateral root proliferation to increase soil exploration (Kuzyakov and Razavi, 2019). These adaptive responses are largely coordinated through auxin signaling networks, which integrate environmental signals with developmental processes. A mechanistic study on the response of *Broussonetia papyrifera* to cadmium contamination by Li et al. (2024) hypothesized that auxin is the driver of tolerance observed. In addition to endogenous auxin biosynthesis within plants, exogenous auxin can influence root development (Yu et al., 2022). This is particularly so for microbial auxin within the rhizosphere. Microbial production of auxin in the rhizosphere alters auxin gradients around plant roots thus, integrating with endogenous plant hormone pathways to influence gene expression and hormonal signaling (Kuzyakov and Razavi, 2019; Orozco-Mosqueda et al., 2023; Zhang et al., 2025). This cross-kingdom biochemical integration is what drives the significant subsequent changes in RSA. In a review of mechanistic studies, Yu et al. (2022) indicated that plants have evolved to have plethora of auxin receptors to capture any signaling. As such, under stressful environmental conditions, sustained auxin signaling may improve plants’ ability to absorb water and nutrients through modified RSA. Understanding the mechanisms through which auxin regulates RSA provides a conceptual framework for exploring how auxin-producing rhizosphere microorganisms influence plant growth through hormone-mediated interactions. This connection forms the basis for investigating the role of auxin in plant–microbiome communication within the rhizosphere.

## 3 Auxin production by rhizosphere microorganisms

Microbial biosynthesis of indole-3-acetic acid (IAA) represents one of the most widely distributed and extensively studied mechanisms through which rhizosphere microbial communities modify root morphology and stimulate host development (Cai et al., 2022; Elsoud et al., 2023; Kangsopa et al., 2025a; Khianngam et al., 2023; Tang et al., 2023). This hormonal trait is highly conserved across diverse taxonomic groups, spanning both bacterial (including *Pseudomonas* (Uzma et al., 2022), *Bacillus* (Kangsopa et al., 2025a), *Azospirillum* (Tang et al., 2023), *Rhizobium* (Kang and Seo, 2022), *Enterobacter* (Kangsopa et al., 2025a)) and fungal genera (include *Funneliformis* (Liu et al., 2024) and Trichoderma (Wang et al., 2025)). These microorganisms typically produce auxin through tryptophan-dependent metabolic pathways, in which tryptophan released from plant root exudates serves as a precursor for microbial IAA biosynthesis (Elsoud et al., 2023). In the tryptophan-dependent pathways of microorganisms, there are different intermediate metabolites with recent studies classifying them into five pathways: the indole-3-acetamide pathway (IAM), the indole-3-pyruvic acid pathway (IPA/IPyA), the indole-3-acetonitrile pathway (IAN), the tryptamine pathway (TAM) and the tryptophan side-chain oxidase pathway (TSO) (Tang et al., 2023). The presence of these metabolic pathways allows rhizosphere microbes to convert plant-derived substrates into biologically active auxin molecules.

Auxin produced by rhizosphere microbes exerts significant effects on plant development, particularly on the formation of root structures (Mal and Panchal, 2024; Xu et al., 2023). Beyond directly improving a plant’s exploratory capacity for soil resources (Gupta et al., 2023), microbial auxin production also plays an important role in shaping broader plant–microbe ecological interactions in the rhizosphere. Auxin can influence microbial colonization (Chen and Liu, 2024) patterns by modifying root exudation profiles and root surface characteristics, which in turn affect the recruitment of beneficial microorganisms (Martins et al., 2026; Xu et al., 2023). Through these processes, stabilized mutualistic feedback loops are created. Similarly, inoculating plants with *R. irregularis* alongside exogenous auxin improved root production amidst heavy metal contamination (Li et al., 2024; Pons et al., 2020).

## 4 Auxin-mediated plant–microbiome communication

An important area of current research is determining how endogenous auxin homeostasis interacts with microbial-derived phytohormones and the extent to which plants can modulate or buffer these external signals. Hardline physiological perspectives frequently argue that internal regulatory checkpoints, such as the rapid degradation kinetics of AUX/IAA repressor proteins and the tight feedback loops of PIN efflux carriers, are evolved to maintain strict morphological homeostasis (Taiz et al., 2015). Under this view, minor localized influxes of microbial IAA should be effectively neutralized or sequestered by the host to prevent developmental chaos. However, some primary studies established that exogenous auxin influences the trajectory of lateral root development (Ivanchenko et al., 2010; Li et al., 2024). Under severe abiotic stresses (such as drought, salinity or nutrient deficiency) host plant’s endogenous auxin biosynthesis pathways are frequently suppressed or spatially disrupted due to systemic metabolic limitations (Zinta et al., 2022). It is precisely within this window of vulnerability that microbial IAA serves as an exogenous rescue mechanism. Rather than parasitically hijacking the host system, beneficial rhizobacteria exploit existing host perception pathways to initiate structural remediation that the stressed plant cannot energetically coordinate on its own (Orozco-Mosqueda et al., 2023; Zhang et al., 2025).

A complex biochemical communication and interactive network exists in the rhizosphere where plants and microorganisms interact (Taiz et al., 2015). Within this ecological network, Auxin plays a significant role (Iqbal et al., 2023; Rawat and Laxmi, 2025). As illustrated in the ARRA framework (**Figure 1**), this interaction is actively driven by plant root exudates, which dictate the composition and stability of the surrounding microbial community (Ma et al., 2022; Tang et al., 2023). These exudates provide nutrients for soil microorganisms and act as chemical signals that influence microbial community composition and stability (Issifu et al., 2025). Certain root-derived compounds, such as flavonoids and organic acids, can selectively stimulate the growth of auxin-producing microorganisms in the rhizosphere (Martins et al., 2026; Orozco-Mosqueda et al., 2023; Xu et al., 2025).

**Figure 1:**
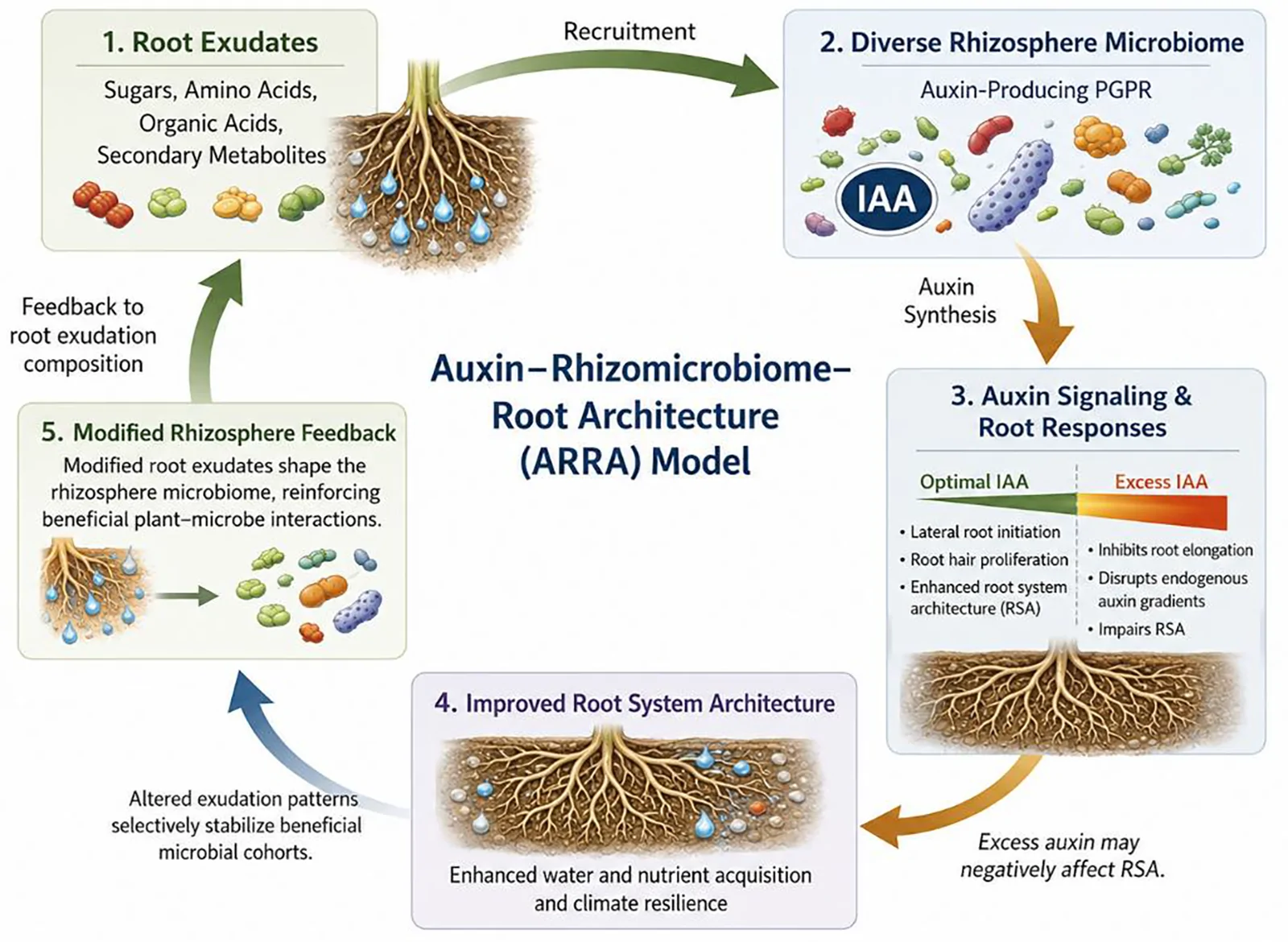
Conceptual illustration of the ARRA framework showing the bidirectional interactions between plant root exudates, auxin-producing rhizosphere microorganisms, and root system architecture under abiotic stress conditions.

Importantly, the integration of auxin-mediated signaling with rhizosphere microbiome engineering represents a promising frontier in plant science, bridging fundamental biology with applied agricultural innovation. The response to the exudates by plant roots is a production of bioactive metabolites by rhizosphere microorganisms that affect plant physiology (Timofeeva et al., 2024). Auxin produced by rhizosphere microbes can alter plant gene expression by interacting with plant auxin signaling pathways (Gupta et al., 2023). This interaction may influence processes such as cell division, root branching and root hair development, which modify the RSA and ultimately resulting into above ground responses such as drought tolerance (Mal and Panchal, 2024; Ranjan et al., 2024; Xu et al., 2023; Zhang et al., 2025).

The synthesis of the Auxin–Rhizomicrobiome–Root Architecture (ARRA) model highlights a profound paradigm shift in our understanding of rhizosphere dynamics under environmental stress. Historically, plant–microbe interactions have been viewed through a fragmented lens, separating host developmental biology from microbial ecology. The ARRA model bridges this gap, establishing that microbially derived indole-3-acetic acid (IAA) acts not merely as an accidental metabolic byproduct, but as a primary cross-kingdom signaling product that dynamically reconstruct host root architecture to secure climate resilience (Iqbal et al., 2023; Rawat and Laxmi, 2025).

Crucially, the success of this symbiotic reprogramming relies entirely on a concentration-dependent tipping point, as visually presented in the ARRA framework (**Figure 1**). The transition from optimal stimulation to supra-optimal inhibition underscores a delicate evolutionary balance in the rhizosphere:

- The stimulatory window: Low, localized flux spikes trigger targeted pericycle cell divisions, optimizing the root system architecture (RSA) for moisture and nutrient exploration (Enders and Strader, 2015). Working on Arabidopsis, Ivanchenko et al. (2010) reported stimulatory gradient of up to 5 nM, above which inhibition initiates. With a soil medium, the concentration may be higher (Li et al., 2024).
- The inhibitory threshold: Conversely, unmanaged or excessive auxin accumulation of over 25 nM overrides endogenous elongation gradients, resulting in primary root stunting and impaired RSA (Ivanchenko et al., 2010).

This strict concentration dependency explains why single-strain inoculants often yield highly variable results in open field trials, where soil moisture, texture, and microbial degradation rates drastically alter localized hormone concentrations. Furthermore, as mapped in our hormonal crosstalk network (**Table 1**), this exogenous auxin influx must be successfully integrated with endogenous stress-signaling pathways like ABA and ethylene to prevent physiological toxicity (Jing et al., 2023; Mal and Panchal, 2024).

Consequently, this continuous loop of the plant influencing microbial characteristics in the rhizosphere is the functional core of the ARRA model. By integrating these cross-kingdom signals, the framework illustrates how adaptive RSA traits are programmed to secure crop resilience. Crucially, the model accounts for concentration-dependent host responses, acknowledging that while balanced microbial IAA inputs improve root branching, an excess of exogenous auxin may negatively affect RSA by overriding endogenous elongation gradients. This bidirectional exchange establishes a stabilized feedback loop where adaptive architectural modifications continually optimize exudation patterns to reinforce beneficial microbial recruitment under stress.

The final architectural modification of the root system does not represent the end of the signaling cascade, but rather the stabilization of a long-term survival loop. An altered root system architecture fundamentally changes the physical and chemical microenvironment of the soil matrix. Expanded root surfaces alter mechanical impedance, increase localized exudation zones, and drive physical rhizosheath formation (Xu et al., 2023). This structural modification alters subsequent root exudation signatures, strategically selecting for and reinforcing the very microbial cohorts capable of producing balanced auxin inputs (Chen and Liu, 2024; Martins et al., 2026). Ultimately, this bidirectional feedback loop establishes a self-sustaining ecological niche that maintains plant physiological functionality and secures crop productivity under intense environmental pressure.

## 5 Auxin-driven rhizomicrobiome effects on abiotic stress tolerance

Global agricultural environment is increasingly under pressure from a lot of stress factors such as drought, heat, salinity, metal toxicity etc. These stress factors can severely impair plant growth by disrupting physiological processes and limiting resource availability. Plant–microbiome interactions mediated by auxin play a crucial role in enhancing plant tolerance to environmental stresses (Iqbal et al., 2023; Li et al., 2024; Martins et al., 2026; Timofeeva et al., 2024; Uzma et al., 2022). Auxin-producing rhizobacteria can mitigate these adverse effects by modifying root architecture and improving plant resource acquisition. One of the primary mechanisms through which microbial auxin enhances stress tolerance is by stimulating the development of more extensive root systems. Increased lateral root formation and root hair density expand the root surface area, enabling plants to explore a larger soil volume and access water and nutrients more efficiently. Moreover, some of the organisms enhance the production of antioxidants and osmo-protectants, further improving the tolerance of plant to abiotic stress (Li et al., 2024).

Auxin also interacts with other plant hormones involved in stress signaling, including abscisic acid (ABA), ethylene and cytokinins as depicted in (**Table 1**) (Taiz et al., 2015; Jing et al., 2023). These hormonal interactions regulate physiological responses such as stomatal conductance, antioxidant activity and osmotic adjustment. For instance, the interactions between Auxin and ABA/Jasmonic Acid (JA) is known to improve drought tolerance (Mal and Panchal, 2024). Through these mechanisms, auxin-producing rhizobacteria can indirectly improve plant tolerance to environmental stress by stabilizing plant hormonal balance and enhancing stress-responsive pathways. Furthermore, recent studies demonstrate that microbial auxin plays a direct role in structuring specialized root–soil complexes, such as rhizosheaths. These structures improve soil aggregation, increase mechanical impedance, and enhance water retention around plant roots, directly improving plant adaptation to severe soil drying conditions (Xu et al., 2023, 2025). These structures create a favorable microenvironment that helps maintain root hydration during periods of water scarcity. As a result, auxin-mediated plant–microbiome interactions can significantly improve plant survival and productivity under stressful environmental conditions. However, there is still debate regarding the extent to which microbial auxin production versus plant endogenous auxin controls root architectural responses under stress conditions.

**Table 1:** Interaction of auxin with other plant growth regulators to drive RSA and stress tolerance.

*Hormone*	Synergistic interaction with auxin	Antagonistic interaction with auxin	Reference
*Cytokinins*	i. Promote cell division in the root vascular cambium ii. Signal nutrient gradient	i. Represses auxin transcription during root meristem specification ii. Inhibits lateral root initiation	(Jan et al., 2024; Romanov, 2022)
*Gibberellins*	i. Promotes root cell elongation through destabilization of DELLA ii. Maintains the size of root apical meristem	i. Inhibits lateral root formation ii. Repress the accumulation of IAA in some cells	(Castro-Camba et al., 2022; Hu et al., 2022)
*Abscisic Acid*	i. Upregulates the accumulation of IAA under drought and salinity condition	i. Regulates the transportation of auxin ii. Inhibits lateral roots emergence and growth	(Kangsopa et al., 2025; Liu et al., 2023)
*Ethylene*	i. Promotes root hair elongation ii. Determines root angles	i. Inhibits primary root elongation ii. Suppresses the initiation of new lateral roots	(Jan et al., 2024; Maric, 2024)
*Jasmonic Acid*	i. Promotes lateral root formation ii. Promotes adventitious root formation	i. Inhibits primary root elongation ii. Regulates source-sink dynamics under stress	(Samanta and Roychoudhury, 2025; Umar et al., 2025)
*Salicylic Acid*	i. Promotes root meristematic cell division at low concentration	i. Inhibits lateral and adventitious root development ii. Modulates auxin transportation	(Rawat and Laxmi, 2025; Verma et al., 2025)
*Strigolactones*	i. Inhibits crown root elongation ii. Stimulates root hair elongation and branching	i. Suppresses lateral root formation	(Jan et al., 2024; Thapar et al., 2024; Wu et al., 2022)
*Brassinosteroids*	i. Promotes lateral root development ii. Promotes primary root elongation	i. Reduces root meristem size at high concentration	(Guo et al., 2024; Wu et al., 2024)

## 6 Biotechnological applications, knowledge gap and research priorities

Our understanding of the mechanisms underlying auxin-mediated plant–microbiome interactions provides leading opportunities to develop innovative agricultural technologies. One promising application will be the use of auxin-producing PGPR as targeted bioinoculants to drive robust root system architecture (RSA) development. Preliminary scaling methods, such as bacterial seed-coating formulations, have already demonstrated measurable success in altering early seedling root traits and nutrient acquisition (Kangsopa et al., 2025a). Advances in microbiome engineering could open new possibilities for designing synthetic microbial consortia that optimize plant hormone regulation in the rhizosphere (Ali et al., 2024; Orozco-Mosqueda et al., 2023). By selecting microorganisms with complementary functional traits, researchers can develop microbial consortia capable of enhancing plant growth and stress resilience. Such approaches represent a shift toward microbiome-assisted agriculture, in which beneficial microbial communities are intentionally manipulated to improve crop performance. Basu et al. (2021) reported that there is an increasing demand for PGPR all over the world with market value reaching 3.5 billion USD. Moreover, research is intensifying in discovering improved formulations for microbial consortia (Monica et al., 2025).

Another emerging strategy involves integrating knowledge of microbial auxin production with molecular breeding and genetic engineering approaches. Identifying plant genes involved in auxin perception, transport and signaling may enable the development of crop varieties that respond more efficiently to microbial signals (Liu et al., 2023). Combining plant genetic improvement with microbial biotechnology could therefore create synergistic strategies for sustainable agricultural production.

Despite recent progress, several critical gaps remain in understanding auxin-mediated plant–microbiome interactions. First, the spatial and temporal dynamics of auxin gradients in the rhizosphere are poorly characterized under field conditions (Ali et al., 2024). While hydroponic assays show precision in localized spikes (Ivanchenko et al., 2010), soil medium records higher fluctuations due to obviously soil characteristics (Li et al., 2024). There is a limited understanding of the mechanism of auxin diffusion and stability in soil medium. Second, the quantitative contribution of microbial-derived auxin relative to endogenous plant auxin remains unclear. We understand that plants regulating auxin signaling internally through the Aux/IAA repressor degradation kinetics and polar PIN efflux carrier feedback loop. Under stress condition, these pathways are suppressed (Zinta et al., 2022). We have limited quantitative appreciation of how microbial auxin over-rides the pathway to trigger root development. Moreover, our understanding is limited regarding the mechanism by which plants differentiate between endogenous and microbial exogenous auxin, especially in crosstalk with other stress hormones. Third, the specificity of plant–microbe signaling across different crop systems and environmental conditions requires further investigation (Liu et al., 2023). While certain root-derived compounds, such as specific flavonoids and organic acids, are known to selectively stimulate and recruit auxin-producing microorganisms (Martins et al., 2026; Xu et al., 2025), this recruitment strategy is highly host-specific and variable (Ivanchenko et al., 2010). To overcome these challenges future research should prioritize multi-omics approaches, including metagenomics, transcriptomics, and metabolomics, to elucidate the regulatory networks governing auxin-mediated interactions. In addition, field-based validation of microbiome-assisted strategies is essential to translate laboratory findings into practical agricultural applications.

## 7 Future perspectives

To advance the Auxin–Rhizomicrobiome–Root Architecture (ARRA) model beyond its current conceptual boundaries, future research must shift toward high-precision synthetic biology and long-term ecological forecasting. A primary horizon lies in deploying CRISPR-Cas genome editing to engineer “microbiome-optimized” crop varieties (Liu et al., 2023). Instead of relying solely on traditional breeding, targeted modifications can fine-tune the sensitivity of host auxin receptors (such as the TIR1/AFB F-box proteins) or alter the expression kinetics of PIN-formed (PIN) efflux carriers (Taiz et al., 2015). By creating plants with localized hypersensitivity to microbially derived indole-3-acetic acid (IAA), it may be possible to maximize lateral root branching and root hair proliferation without triggering the negative, growth-inhibiting physiological effects associated with systemic, supra-optimal auxin accumulation (Orozco-Mosqueda et al., 2023).

Another critical frontier is the evolutionary stability of synthetic microbial consortia (SynComs) in open, competitive agronomic systems (Ke et al., 2021). While multi-strain bioinoculants frequently show robust initial efficacy in sterile or controlled settings, the long-term persistence of engineered hormonal traits across successive plant generations remains a major challenge. Soil microbiomes are highly competitive environments where non-producing “cheater” strains often outcompete engineered auxin-producers over time. To counter this, future studies must design self-stabilizing SynComs linked by obligate metabolic dependencies, ensuring that microbial auxin synthesis is energetically and functionally coupled with the utilization of specific, host-exclusive root exudates (Chen and Liu, 2024; Finkel et al., 2020).

Finally, the ARRA framework should be explicitly integrated into global climate-mitigation strategies. Optimizing root system architecture (RSA) for severe environmental stress tolerance simultaneously drives deep-root biomass expansion and physical rhizosheath development (Xu et al., 2023, 2025). This coordinated structural modification directly enhances the deposition and stabilization of recalcitrant organic carbon compounds into deeper soil horizons, effectively turning modified crop root systems into highly functional sinks for long-term atmospheric carbon sequestration (Kuzyakov and Razavi, 2019). Linking rhizosphere hormonal dynamics to macro-level carbon accounting represents a vital, untapped bridge between molecular plant physiology and sustainable global agriculture.

## 8 Conclusion

The synthesis of the Auxin–Rhizomicrobiome–Root Architecture (ARRA) model confirms that auxin serves as a critical, bidirectional cross-kingdom signaling currency between host plants and rhizosphere microorganisms. Many rhizospherePGPR produce auxin, which influences host hormonal signaling pathways and modifies root morphology. These changes enhance soil exploration and contribute to plant adaptation under abiotic stress conditions. Moreover, changes in the RSA establish a self-sustaining feedback loop by altering subsequent root exudation profiles to selectively recruit beneficial microbial consortia. The efficacy, however, remains strictly governed by the concentration of auxin produced; flowing from root stimulation to inhibition as concentration increases. Ultimately, the integration of plant physiology, microbial ecology, molecular biology and agricultural biotechnology provides new opportunities for harnessing auxin-mediated plant–microbiome interactions to enhance sustainable agriculture. Strategies such as microbial inoculants, microbiome engineering and hormone-responsive crop breeding will play an important role in developing climate-resilient cropping systems. Continued research in these areas is essential to unlock the full potential of the rhizosphere microbiome in supporting global food security.
